# Cardiopulmonary Bypass Time During Surgery for Acute Type A Aortic Dissection and Mid-Term Survival

**DOI:** 10.3390/jcdd12040139

**Published:** 2025-04-07

**Authors:** Mikko Uimonen, Christian Olsson, Anders Jeppsson, Arnar Geirsson, Vibeke Hjortdal, Emma C. Hansson, Igor Zindovic, Jacob Ede, Jarmo Gunn, Anders Wickbom, Tomas Gudbjartsson, Ari Mennander

**Affiliations:** 1 Tampere University Hospital, Heart Hospital, 33520 Tampere, Finland; mikko.uimonen@sydansairaala.fi; 2 Faculty of Medicine and Health Technology, Tampere University, 33520 Tampere, Finland; 3 Karolinska University Hospital, SE-17176 Stockholm, Sweden; christian.olsson@ki.se; 4 Department of Molecular and Clinical Medicine, Institute of Medicine, Sahlgrenska Academy, University of Gothenburg, SE-41345 Gothenburg, Sweden; anders.jeppsson@vgregion.se (A.J.); emma.hansson@vgregion.se (E.C.H.); 5 Department of Cardiothoracic Surgery, Sahlgrenska University Hospital, SE-41345 Gothenburg, Sweden; 6 Division of Cardiac Surgery, Yale School of Medicine, New Haven, CT 06520, USA; arnargeirsson@yahoo.com; 7 Aarhus University Hospital, DK-8200 Aarhus, Denmark; vibeke.hjortdal@regionh.dk; 8 Department of Cardiothoracic Surgery, Clinical Sciences, Skåne University, Lund University, 22100 Lund, Sweden; igor.zindovic@med.lu.se (I.Z.); jacob.ede@med.lu.se (J.E.); 9 Turku University Hospital, 20521 Turku, Finland; jarmo.gunn@tyks.fi; 10 Orebro University Hospital, 70116 Orebro, Sweden; anders.wickbom@gmail.com; 11 Department of Cardiothoracic Surgery, Landspitali University Hospital, Faculty of Medicine, University of Iceland, 101 Reykjavik, Iceland; tomasgudbjartsson@hotmail.com

**Keywords:** acute type A aortic dissection, duration of cardiopulmonary time, survival

## Abstract

We evaluated the association between cardiopulmonary bypass (CPB) time during surgery for acute type A aortic dissection (ATAAD) and mid-term survival. Data of 1122 patients who underwent surgery for ATAAD in eight Nordic centers from January 2005 to December 2014 were retrospectively analyzed. An adjusted logistic regression analysis was performed to investigate the association of incremental 30 min CPB time on 30-day mortality. In addition, the patients were divided into those that underwent surgery with >210 min (n = 369) or <210 min CPB time (n = 605) based on spline analysis and a receiver operating characteristic curve. The restricted mean survival time ratios adjusted for patient characteristics and surgical details between the groups were calculated for survival and aortic reoperation-free survival. The median follow-up time was 2.6 (inter-quartile range 0.9–4.9) years. Incremental CPB time was associated with higher 30-day mortality (OR 1.25 per 30 min, 95% CI 1.15–1.35, *p* < 0.001). Mid-term survival for all patients was inferior in the >210 min group as compared with the <210 min group (adjusted restricted mean survival time ratio 0.88, 95% confidence interval [CI] 0.81–0.96, *p* = 0.003). Reoperation-free survival was similar in patients with CPB time > 210 min as compared with <210 min. Prolonged CPB time is associated with higher 30-day mortality and inferior mid-term survival but not with inferior reoperation-free survival after surgical repair of ATAAD.

## 1. Introduction

Acute type A aortic dissection (ATAAD) is a complex cardiovascular event associated with high mortality. In a population-based study, it was found that almost half of ATAAD patients die before reaching the hospital, and early mortality is up to 47.5% for patients that reach the hospital alive [1].

The immediate aim of surgery for ATAAD is to prevent fatal aortic rupture and cardiac tamponade and to secure blood circulation to the heart and essential organs such as the brain. Surgically, the simplest procedure is the replacement of the supracommissural ascending aorta, but more extensive aortic surgery involving the aortic root and the aortic arch is sometimes required [2]. In addition, concomitant coronary artery bypass grafting and valve surgery may be required, depending on the complexity of the acute disease. Furthermore, the preferences of the surgeon and the possible unplanned events at surgery such as bleeding, myocardial ischemia and valve regurgitation influence the procedure. The extent of aortic repair required has been a matter of extensive discussion [3,4,5]. The decision to limit or choose extended aortic repair is a compromise between increasing the chance of immediate survival and relieving malperfusion, but this is at the possible expense of increasing the risk of late complications including reoperations.

In all complex surgical procedures, the duration of surgery is a common denominator for many technical and patient-related factors that must be considered during ATAAD repair. Previous studies, including aortic surgery, suggest that CPB time influences early outcome, including 30-day mortality [6,7,8,9,10,11]. Although survival after surgery for ATAAD is improving in general, the association of duration of surgery on early and mid-term survival and the incidence of reoperations are less well studied [12]. This study therefore aims to examine the association of CPB time with outcome in ATAAD patients using the Nordic Consortium for Acute Type A Aortic Dissection (NORCAAD) registry. We hypothesize that CPB time for ATAAD, which reflects the surgical strategy in patients with ATAAD but also the extent and severity of the dissection and perioperative complications, is associated with 30-day mortality, mid-term survival and reoperation-free survival.

## 2. Materials and Methods

### 2.1. Ethical Statement

The institutional review boards of each participating center have approved the study protocol. Locally, the study was approved by the Research Ethics Committee of Tampere (ETL R15504/1.1.2015). Due to the retrospective nature of the study, the need for informed consent was waived.

### 2.2. Patients

The NORCAAD project involves eight tertiary care cardiac surgery units in four Nordic countries (Denmark, Finland, Iceland and Sweden). The NORCAAD register encompasses all patients that have undergone surgery for ATAAD in the study hospitals within the study period from 1 January 2005 until 31 December 2014, resulting in a total of 1122 patients with onset of symptoms leading to surgery for ATAAD in less than 2 weeks; eighty-three per cent of the patients had symptoms in less than 48 h before surgery (Supplementary Figure S1). A detailed description of the NORCAAD register has been published previously [13].

Altogether, 974 patients with recorded CPB time and complete follow-up data were included in the analysis. CPB time was determined as the total time spent from the onset of CPB till the end of weaning from CPB. There were 148 (13.2%) patients with missing data on CPB (n = 98, 8.8%) or follow-up (n = 50, 4.5%).

### 2.3. Main Outcome Measures

The primary endpoints were 30-day mortality and mid-term survival. The secondary endpoints were perioperative stroke and aortic reoperation-free survival. The definition of postoperative stroke encompassed new neurological symptoms after surgery associated with cerebral infarction or intracerebral hemorrhage. Aortic reoperation was defined as any late open aortic or aortic valve surgery after the index hospitalization.

### 2.4. Statistical Methods

Continuous variables are reported as means and standard deviations (SDs) or medians and inter-quartile ranges when non-normally distributed. Associations of CPB time with 30-day mortality, reoperation and postoperative stroke were examined by unadjusted univariable logistic regression analyses and multivariable logistic regression analyses adjusted by potential confounding variables. The covariates were selected according to previous knowledge of causal relationships (Supplementary Figure S2) and included aortic root surgery (yes/no), aortic arch surgery (yes/no), open distal anastomosis (yes/no), use of antegrade/retrograde cerebral perfusion (yes/no), additional coronary artery bypass (yes/no), ongoing antithrombotic medication (yes/no), previous cardiac surgery (yes/no), patient age (continuous) and body mass index (BMI; continuous). Odds ratios (OR) for each outcome were reported for every 30 min increase in CPB time.

To identify factors associated with extended CPB time, the patients were divided into two groups according to probability of 30-day mortality by CPB time using restricted cubic spline analysis [14]. The culprit cut-off time point of increased 30-day mortality was determined by maximizing the Youden index after spline smoothing, and a receiver operating characteristic curve was performed [15]. Patient characteristics, clinical findings and surgical details were compared between the groups using an independent samples *t*-test for continuous variables and a chi-squared test for categorical variables. Kaplan–Meier curves presenting survival and reoperation-free survival were calculated for the groups. The stability of the Kaplan–Meier estimates was ensured by restricting the follow-up time at the last year with the number of patients at risk including over 10% of the total patient count in the groups, resulting in a maximum follow-up time of 6 years. The groups were compared by a log-rank test. Further, restricted mean survival time (RMST) estimates along with 95% confidence intervals (CI) were calculated for the 6-year follow-up time and compared between the CPB time groups [16,17]. The RMST estimate represents population averages of the amount of event-free survival time during the follow-up period. Crude and adjusted estimates were calculated. Covariates included in the adjusted analyses were the same as in the logistic regression. Subgroup analyses were separately performed among patients with only ascending aortic surgery and patients with only aortic root surgery. A *p*-value < 0.05 was considered statistically significant. Statistical analyses were performed using R (4.0.3) statistical software.

## 3. Results

### 3.1. Patients

The majority of the patients were male (67.4%), and the mean age was 61.0 (SD 12.1) years (Table 1).

The median follow-up time was 2.7 (IQR 0.6–5.3) years. The total accumulated follow-up was 3169 patient-years with a minimum follow-up of one day and a maximum of 10 years. In 681 (69.9%) patients, surgery involved the ascending aorta only (Table 2).

The aortic root was operated on in 243 (24.9%) patients, whereas surgery included total aortic arch replacement in 51 (5.2%) patients. The median CPB time was 189 (IQR 155–234) min (Table 2 and Supplementary Figure S3).

According to the adjusted logistic regression analyses (Table 3 and Supplementary Figure S4), CPB time was associated with increased 30-day mortality (OR 1.25 per 30 min, 95% CI 1.15–1.35, *p* < 0.001) and postoperative stroke (OR 1.17 per 30 min, 95% CI 1.08–1.26, *p* < 0.001, Table 3).

In patients with ascending aortic surgery only and in patients with aortic root surgery only, incremental CPB time was also associated with increased 30-day mortality (OR 1.31 per 30 min, 95% CI 1.17–1.48, *p* < 0.001 and OR 1.17 per 30 min, 95% CI 1.01–1.36, *p* = 0.040), respectively. In patients with ascending aortic surgery only, CPB time was also associated with postoperative stroke (OR 1.19 per 30 min, 95% CI 1.07–1.32, *p* = 0.001) but not in patients with aortic root surgery only (OR 1.16 per 30 min, 95% CI 0.99–1.36, *p* = 0.072).

**Figure 1:**
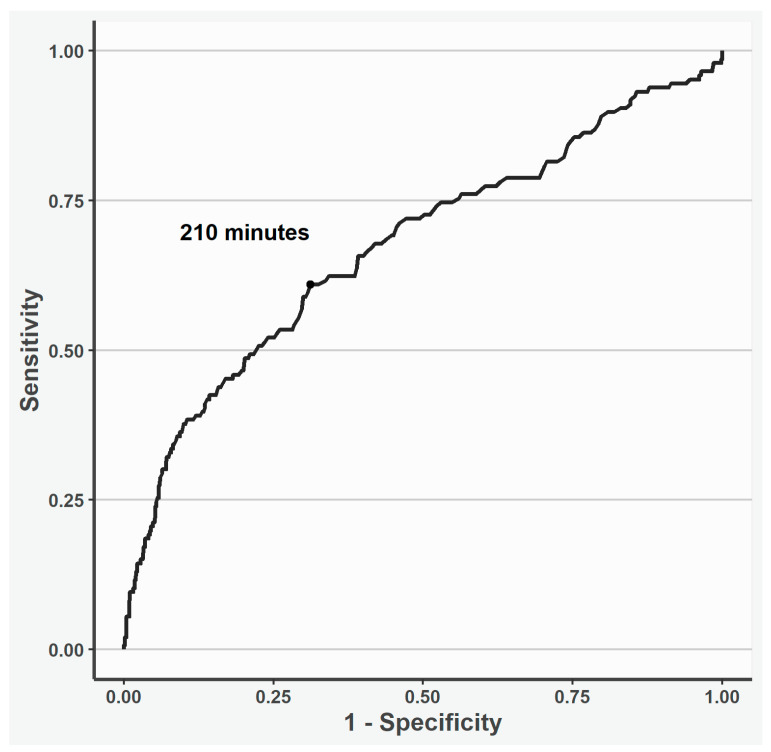
Receiver operating curve of the cut-off cardiopulmonary time point investigated using restricted cubic spline analysis. According to the spline analysis, 210 min was estimated as the cut-off cardiopulmonary time point; a longer time was associated with 30-day mortality.

According to the spline analysis, 210 min was estimated as the cut-off time point, and a longer CPB time was associated with higher 30-day mortality (Figure 1).

CPB time exceeded 210 min in 369 (37.9%) patients, whereas it remained less than 210 min in the remaining patients (n = 605, 62.1%, Table 4).

As expected, duration of surgery, aortic cross-clamp and hypothermic arrest times were increased in patients with CPB time > 210 min as compared with the <210 min group (medians: 439 min vs. 292 min, *p* < 0.001; 141 min vs. 77 min, *p* < 0.001 and 30 min vs. 25 min, *p* < 0.001, respectively). Among the patients with CPB time > 210 min, the proportion of male patients was higher (74.5% vs. 63.0%, *p* < 0.001), BMI was higher (27.4 vs. 26.3, *p* = 0.001), the use of antithrombotic drugs was more frequent preoperatively, both aspirin (30.6% vs. 23.0%, *p* = 0.010) and warfarin (7.9% vs. 4.3%, *p* = 0.028) were used, previous cardiac surgeries were more common (3.8% vs. 0.7%; *p* = 0.001), and aortic dissection was more commonly classified as DeBakey type I (77.2% vs. 70.4%, *p* = 0.044) as compared with patients in the <210 min group. The patients with CPB time > 210 min more often had aortic root surgery as compared with the <210 min group (n = 84, 37.9% vs. n = 140, 13.9%, *p* < 0.001). Total arch surgery (9.2% vs. 2.8%, *p* < 0.001) and concomitant coronary artery bypass grafting were more frequent in the >210 min surgery patients than in the <210 min patients (11.4% vs. 2.8%, *p* < 0.001).

### 3.2. Mid-Term Survival and Reoperation-Free Survival

**Figure 2:**
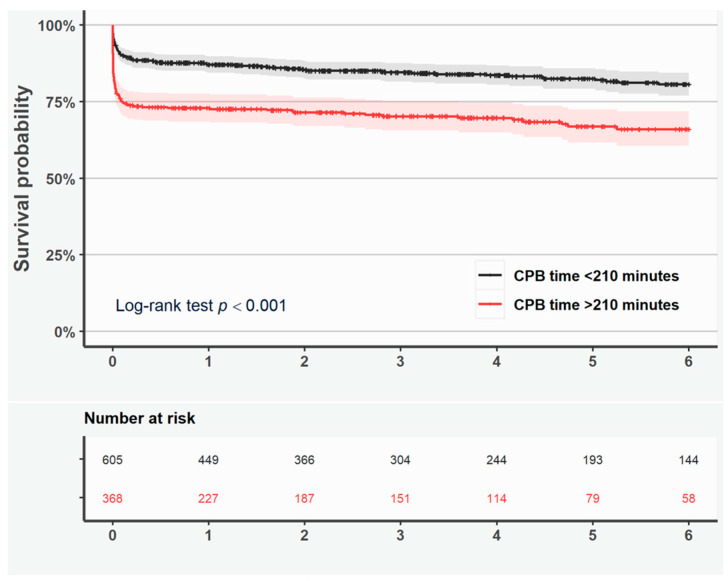
Kaplan–Meier curves representing survival in all patients with duration of cardiopulmonary bypass <210 min (black line) and >210 min (red line). Shaded area represents the 95% confidence interval.

The unadjusted Kaplan–Meier curves showed shorter cumulative survival if CPB time was >210 min (log-rank test *p* < 0.001, Figure 2).

Likewise, the adjusted RMST ratio (0.88, 95% CI 0.81–0.96, *p* = 0.003) indicated that CPB time > 210 min was associated with shorter survival (Table 5).

**Figure 3:**
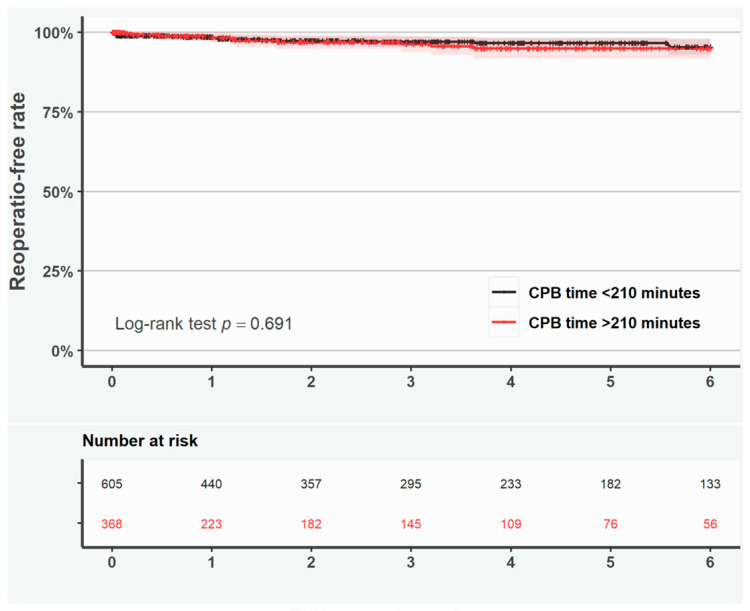
Kaplan–Meier curves representing reoperation-free rate in all patients with duration of cardiopulmonary bypass <210 min (black line) and >210 min (red line). Shaded area represents the 95% confidence interval.

Shorter survival was observed if the duration of surgery exceeded >210 min in patients with ascending aortic surgery only (log-rank test *p* < 0.001; Figure S5), but not in patients with aortic root surgery only (log-rank test *p* = 0.306; Figure S6). Reoperation-free survival was similar in patients with CPB time > 210 min as compared with <210 min according to the Kaplan–Meier curves and adjusted RMST ratio (Figure 3 and Table 5).

## 4. Discussion

The results of the current study demonstrate that CPB time for ATAAD is associated with an increased risk of 30-day mortality, perioperative stroke, and inferior mid-term survival.

CPB time is the product of many factors, both modifiable and non-modifiable. The current study findings indicate that protocol-driven time-consuming surgical strategies should not be implemented routinely but only after careful evaluation of short- and long-term outcomes versus surgical risk. While multifactorial in origin, CPB time appears to be a powerful predictor that could be included in future studies to help define patient and procedural characteristics and analyze outcomes. An improved understanding of the different aspects of the procedural duration associated with outcomes would, however, require a large, contemporary and prospective multi-center study with sufficiently detailed data. Modifiable factors pertinent to the outcome after surgery for ATAAD need to be explored in detail. Such a prospective study is currently underway in the ongoing NORCAAD 2.0 study. Extensive and additional surgical procedures during complex ATAAD repair increase CPB time, but the duration of surgery also reflects the patient’s condition, the extent and severity of the dissection, and perioperative complications including bleeding and malperfusion, as well as the surgeon’s experience [18]. Extensive resection of the aorta and indispensable additional procedures, such as coronary artery bypass grafting, may result in an increased hypothermic circulatory arrest time and/or CPB time, which are both associated with increased 30-day mortality.

By using cubic spline graphs, a cut-off of 210 min was identified to categorize a culprit prolonged CPB time with regards to increased 30-day mortality. Inferior mid-term survival was mainly attributed to the increased 30-day mortality in patients with CPB time > 210 min as compared with <210 min. However, there was no reoperation-free survival difference between the groups, as aortic replacement was performed in the patients according to the extension of the aortic dissection. On the other hand, there were only 5.3% total arch reconstructions, even though 73% of the patients had type I DeBakey ATAAD; long-term follow-up of the patients is warranted.

ATAAD is an emergency requiring immediate surgery in patients with varying pathology and fluctuating symptoms. Indeed, the NORCAAD cohort includes patients with surgery mostly performed in less than 48 h after onset of symptoms. Insufficient knowledge on patient characteristics, underlying aortic wall disease and several comorbidities such as history of stroke, need of coronary artery bypass grafting, previous surgery, ongoing medication, malperfusion and the presence of aortic aneurysm challenge decision making for surgical techniques, cannulation sites, specific cerebral protection methods and the extent of aortic surgery. The risk of respiratory insufficiency, infections, gastrointestinal complications, neurological dysfunction and early mortality increase in patients with prolonged CPB time [8,9,10]. It may be tempting to aim at a one-stage do-it-all reconstructive surgery, at least for a young patient, to avoid reoperations at follow-up. Still, the ultimate strategy should include safe surgery without negatively affecting immediate recovery [19,20,21,22,23].

### Limitations of the Study

The main limitation of the current study is its retrospective design involving a risk of selection bias. Despite the relatively large sample size, only a few patients underwent aortic arch surgery, which ruled out subgroup analysis among these patients. Of the total patients, 13.2% were excluded due to incomplete data on CPB time or follow-up. Though increased CPB time often signifies complex surgery, many additional procedures may be necessary to secure an immediate outcome after ATAAD. Extensive surgery during ATAAD is justified in the presence of complex aortic primary tear dissection beyond the aortic arch and the aortic root and the need for additional surgery due to, e.g., significant coronary artery disease. More detailed data are needed and are currently being collected to understand the modifiable risk factors as opposed to all patient-determined characteristics during ATAAD.

## 5. Conclusions

The effect of patient-dependent and modifiable factors associated with ATAAD need constant evaluation. Prolonged CPB time is associated with increased complexity of ATAAD but also increased risk of 30-day mortality and inferior mid-term survival.

## Figures and Tables

**Table 1:** Patient characteristics.

Characteristics	N = 974
Sex male, n (%)	656 (67.4)
Age, mean (SD)	61.0 (12.1)
BMI, mean (SD)	26.7 (4.7)
Smoking, n (%)	321 (33.9)
Chronic diseases, n (%)	
Hypertension	498 (51.1)
Diabetes mellitus	19 (2.0)
Peripheral vascular disease	27 (2.8)
Hypercholesterolemia	111 (11.4)
Chronic kidney disease	20 (2.1)
Chronic obstructive pulmonary disease	55 (5.6)
Coronary artery disease	37 (3.8)
History of stroke	37 (3.8)
History of TIA	15 (1.5)
Connective tissue disorder, n (%)	
Ehlers-Danlos	1 (0.1)
Marfans	41 (4.2)
Other	5 (0.5)
Antithrombotic medication in use, n (%)	
Aspirin	252 (25.9)
Other antiplatelet agent	110 (11.3)
Warfarin	55 (5.6)
Previously known thoracic aorta aneurysm, n (%)	87 (8.9)
Bicuspid aortic valve, n (%)	53 (5.4)
Family history of aortic dissection, n (%)	52 (5.3)
Family history of thoracic aortic aneurysm, n (%)	36 (3.7)
Previous cardiac surgery, n (%)	18 (1.8)
Previous aortic surgery, n (%)	15 (1.5)
DeBakey class, n (%)	
Type I	711 (73.0)
Type II	263 (27.0)
Preoperative hypotensive shock, n (%)	186 (19.1)
Preoperative cardiac arrest, n (%)	46 (4.7)
Pericardial effusion, n (%)	398 (40.9)
Malperfusion, n (%)	
Any	270 (27.7)
Cardiac	68 (7.0)
Gastrointestinal	27 (2.8)
Renal	53 (5.4)
Iliofemoral vessels	129 (13.2)
Innominate artery	71 (7.3)
Cerebral	68 (7.0)
Spinal	25 (2.6)

**Table 2:** Operation characteristics in all patients.

Proximal repair, n (%)	
Ascending graft only	300 (30.8)
Aortic valve resuspension and ascending graft	398 (40.9)
Aortic valve replacement and ascending graft	24 (2.5)
Biological composite prosthesis	51 (5.2)
Mechanical composite prosthesis	165 (16.9)
Valve sparing conduit prosthesis	30 (3.1)
Unknown/attempted	6 (0.6)
**Distal repair, n (%)**	
Ascending aorta	693 (71.1)
Hemiarch	213 (21.9)
Total arch reconstruction	52 (5.3)
Unknown/attempted	16 (1.6)
**Additional aortic repair, n (%)**	
Aortic root annuloplasty	4 (0.4)
Elephant trunk or frozen elephant trunk	7 (0.7)
Intraluminal graft	3 (0.3)
Suture of layers only	2 (0.2)
Aortic wrapping with mesh	11 (1.1)
Open distal anastomosis, n (%)	840 (86.2)
Cerebral protection technique, n (%)	
Antegrade cerebral perfusion	270 (27.7)
Retrograde cerebral perfusion	227 (23.3)
Hypothermic arrest only	367 (37.7)
Antegrade and retrograde cerebral perfusion	7 (0.7)
Other/unknown	103 (10.6)
Operation time, median (IQR)	330 (277–420)
Cardiopulmonary bypass time, median (IQR)	189 (155–234)
Aortic cross-clamp time, median (IQR)	91 (67–133)
Hypothermic arrest time, median (IQR)	27 (20–36)

**Table 3:** Logistic regression analysis showing associations between duration of cardiopulmonary bypass and early outcome. Odds ratios are presented as 30 min increments in cardiopulmonary bypass time.

	Crude			Adjusted *		
	OR	(95% CI)	*p*	OR	(95% CI)	*p*
**All patients**						
30-day mortality	1.269	(1.195–1.352)	<0.001	1.250	(1.156–1.354)	<0.001
Postoperative stroke	1.160	(1.093–1.231)	<0.001	1.167	(1.082–1.260)	<0.001
**Ascending aortic surgery**						
30-day mortality	1.369	(1.244–1.514)	<0.001	1.314	(1.173–1.475)	<0.001
Postoperative stroke	1.192	(1.093–1.303)	<0.001	1.190	(1.073–1.320)	0.001
**Aortic root surgery**						
30-day mortality	1.234	(1.104–1.388)	<0.001	1.167	(1.007–1.357)	0.040
Postoperative stroke	1.182	(1.047–1.337)	0.007	1.156	(0.988–1.357)	0.072

* Adjusted by root reconstruction, arch reconstruction, open distal anastomosis, selective antegrade cerebral perfusion, additional coronary artery bypass, ongoing antithrombotic medication, previous cardiac surgery, patient age and body mass index. OR = odds ratio, CI = confidence interval.

**Table 4:** Preoperative and operative characteristics according to cardiopulmonary bypass time < 210 min and >210 min.

	Cardiopulmonary Bypass Time		*p*
	<210 min, n = 605	>210 min, n = 369	
**Preoperative characteristics**			
**Sex male, n (%)**	381 (63.0)	275 (74.5)	<0.001
**Malperfusion, n (%)**			
Cardiac	28 (4.6)	40 (10.8)	<0.001
Cerebral	34 (5.6)	34 (9.2)	0.045
Gastrointestinal	11 (1.8)	16 (4.3)	0.034
Renal	37 (6.1)	16 (4.3)	0.297
Iliofemoral vessels	85 (14.0)	44 (11.9)	0.394
Spinal	18 (3.0)	7 (1.9)	0.410
Any	163 (26.9)	107 (29.0)	0.534
Innominate arteries	45 (7.4)	26 (7.0)	0.919
**BMI, mean (SD)**	26.27 (4.57)	27.38 (4.88)	0.001
**Previous cardiac surgery, n (%)**	4 (0.7)	14 (3.8)	0.001
**Antithrombotic medication in use, n (%)**			
Aspirin	139 (23.0)	113 (30.6)	0.010
Warfarin	26 (4.3)	29 (7.9)	0.028
Other antiplatelet agents	60 (9.9)	50 (13.6)	0.102
**Chronic diseases, n (%)**			
Peripheral vascular disease	9 (1.5)	18 (4.9)	0.003
Coronary artery disease	18 (3.0)	19 (5.1)	0.121
Diabetes mellitus	9 (1.5)	10 (2.7)	0.272
Hypertension	302 (49.9)	196 (53.1)	0.367
History of stroke	20 (3.3)	17 (4.6)	0.391
History of TIA	11 (1.8)	4 (1.1)	0.526
Hypercholesterolemia	66 (10.9)	45 (12.2)	0.611
Chronic kidney disease	11 (1.8)	9 (2.4)	0.667
Chronic obstructive pulmonary disease	36 (6.0)	19 (5.1)	0.702
**DeBakey, n (%)**			0.044
Type I	426 (70.4)	285 (77.2)	
Type II	179 (29.6)	84 (22.8)	
**Smoking, n (%)**	189 (31.4)	132 (35.8)	0.065
**Pericardial effusion, n (%)**	234 (38.7)	164 (44.4)	0.087
**Preoperative cardiac arrest, n (%)**	23 (3.8)	23 (6.2)	0.114
**Family history for thoracic aortic aneurysm, n (%)**	18 (3.0)	18 (4.9)	0.176
**Preoperative hypotensive shock, n (%)**	108 (17.9)	78 (21.1)	0.237
**Previously known thoracic aorta aneurysm, n (%)**	50 (8.3)	37 (10.0)	0.412
**Connective tissue disorder, n (%)**			0.507
Marfans	23 (3.8)	18 (4.9)	
Ehlers-Danlos	1 (0.2)	0 (0.0)	
Other	2 (0.3)	3 (0.8)	
**Age, mean (SD)**	61.16 (12.19)	60.85 (11.95)	0.695
**Bicuspid aortic valve, n (%)**	32 (5.3)	21 (5.7)	0.902
**Family history for aortic dissection, n (%)**	33 (5.5)	19 (5.1)	0.953
**Previous aortic surgery, n (%)**	9 (1.5)	6 (1.6)	1.000
**Operative characteristics**			
**Coronary artery bypass grafting, n (%)**	17 (2.8)	42 (11.4)	<0.001
**Aortic cross clamp time, minutes, median (IQR)**	77 (59–99)	141 (99–178)	<0.001
**Hypothermic arrest time, minutes, median (IQR)**	25 (19–33)	30 (22–41)	<0.001
**Proximal repair, n (%)**			<0.001
Ascending graft only	202 (33.4)	98 (26.6)	
Aortic valve resuspension and ascending graft	301 (49.8)	97 (26.3)	
Aortic valve replacement and ascending graft	6 (1.0)	18 (4.9)	
Biological conduit prosthesis	11 (1.8)	40 (10.8)	
Mechanical conduit prosthesis	70 (11.6)	95 (25.7)	
Valve sparing conduit prosthesis	9 (1.5)	21 (5.7)	
Unknown	6 (1.0)	0 (0.0)	
**Distal repair, n (%)**			0.001
Ascending aorta	440 (72.7)	253 (68.6)	
Hemiarch	136 (22.5)	77 (20.9)	
Total arch	18 (3.0)	34 (9.2)	
Unknown	11 (1.9)	5 (1.3)	
**Open distal anastomosis, n (%)**	507 (83.8)	333 (90.2)	0.006
**Additional aortic repair, n (%)**			0.057
Aortic root annuloplasty	2 (0.3)	2 (0.5)	
Elephant trunk or frozen elephant trunk	1 (0.2)	6 (1.6)	
Intraluminal graft	3 (0.5)	0 (0.0)	
Suturing of aortic layers	2 (0.3)	0 (0.0)	
Aortic wrapping with mesh	5 (0.8)	6 (1.6)	
**Cerebral protection technique, n (%)**			0.217
Antegrade cerebral perfusion	229 (37.9)	138 (37.4)	
Retrograde cerebral perfusion	158 (26.1)	112 (30.4)	
Hypothermic arrest only	139 (23.0)	88 (23.8)	
Antegrade and retrograde cerebral perfusion	4 (0.7)	3 (0.8)	
Other/unknown	75 (12.4)	28 (7.6)	

**Table 5:** RMST ratio estimates representing survival and reoperation-free survival in patients with duration of surgery < 210 min and >210 min.

	Crude				Adjusted *	
	RMST (95% CI)		RMST Ratio ** (95% CI)	*p*	RMST Ratio ** (95% CI)	*p*
	<210 min	>210 min				
**All patients**						
Survival	5.08 (4.91–5.25)	4.22 (3.94–4.49)	0.83 (0.77–0.89)	<0.001	0.88 (0.81–0.96)	0.003
Reoperation-free survival	5.73 (5.63–5.83)	5.76 (5.62–5.83)	1.00 (0.98–1.03)	0.790	0.99 (0.97–1.02)	0.670
**Ascending aortic surgery**						
Survival	5.21 (5.03–5.38)	4.20 (3.81–4.58)	0.81 (0.73–0.89)	<0.001	0.84 (0.76–0.93)	0.001
Reoperation-free survival	5.74 (5.63–5.85)	5.81 (5.65–5.97)	1.01 (0.98–1.05)	0.478	1.00 (0.97–1.03)	0.900
**Aortic root surgery**						
Survival	4.65 (4.11–5.18)	4.34 (3.90–4.78)	0.93 (0.80–1.09)	0.387	0.99 (0.83–1.19)	0.910
Reoperation-free survival	5.72 (5.43–6.00)	5.71 (5.46–5.96)	1.00 (0.94–1.07)	0.977	1.07 (0.98–1.17)	0.121

* Adjusted by occurrence of root reconstruction, arch reconstruction, open distal anastomosis, use of cerebral perfusion, additional coronary artery bypass, ongoing antithrombotic medication, previous cardiac surgery, patient age and body mass index. ** >210 min/<210 min, CI = confidence interval, RMST = restricted mean survival time.

## Data Availability

All authors had full access to all of the data in the study and take responsibility for the integrity of the data and the accuracy of the data analysis. The data underlying this article will be shared upon reasonable request to the corresponding author.

## Supplementary Materials

The following supporting information can be downloaded at https://www.mdpi.com/article/10.3390/jcdd12040139/s1, Supplementary Figure S1: Distribution of delay from symptom onset to surgery according to patients with duration of cardiopulmonary bypass <210 min (black box) and >210 min (red box); Supplementary Figure S2: Causal relationship diagram showing rationale for covariate selection; Supplementary Figure S3: Distribution of cardiopulmonary bypass time; Supplementary Figure S4: Association between cardiopulmonary time and outcome. Lines show the probability of the outcome event with regards to cardiopulmonary time. Gray area represents 95% confidence interval. Histograms show the distributions of cardiopulmonary time with early mortality or postoperative stroke (100% probability) vs. not (0 probability). Figure S5: Kaplan–Meier curves representing survival in patients with ascending aortic surgery only with duration of cardiopulmonary bypass <210 min (black line) and >210 min (red line). Shaded area represents the 95% confidence interval. Figure S6: Kaplan–Meier curves representing survival in patients with aortic root surgery only with duration of cardiopulmonary bypass <210 min (black line) and >210 min (red line). Shaded area represents the 95% confidence interval.

## Abbreviations

The following abbreviations are used in this manuscript:

ATAAD	Acute type A aortic dissection
BMI	Body mass index
CI	Confidence interval
CPB	Cardiopulmonary bypass
IQR	Inter-quartile range
OR	Odds ratios
NORCAAD	Nordic Consortium for Acute Type A Aortic Dissection
RMST	Restricted mean survival time
SD	Standard deviation
