# Supplementary figures and images for: Cardiopulmonary Bypass Time During Surgery for Acute Type A Aortic Dissection and Mid-Term Survival
Source: J Cardiovasc Dev Dis. 2025 Apr 7;12(4):139. doi: 10.3390/jcdd12040139 (PMC12028012; doi:10.3390/jcdd12040139)

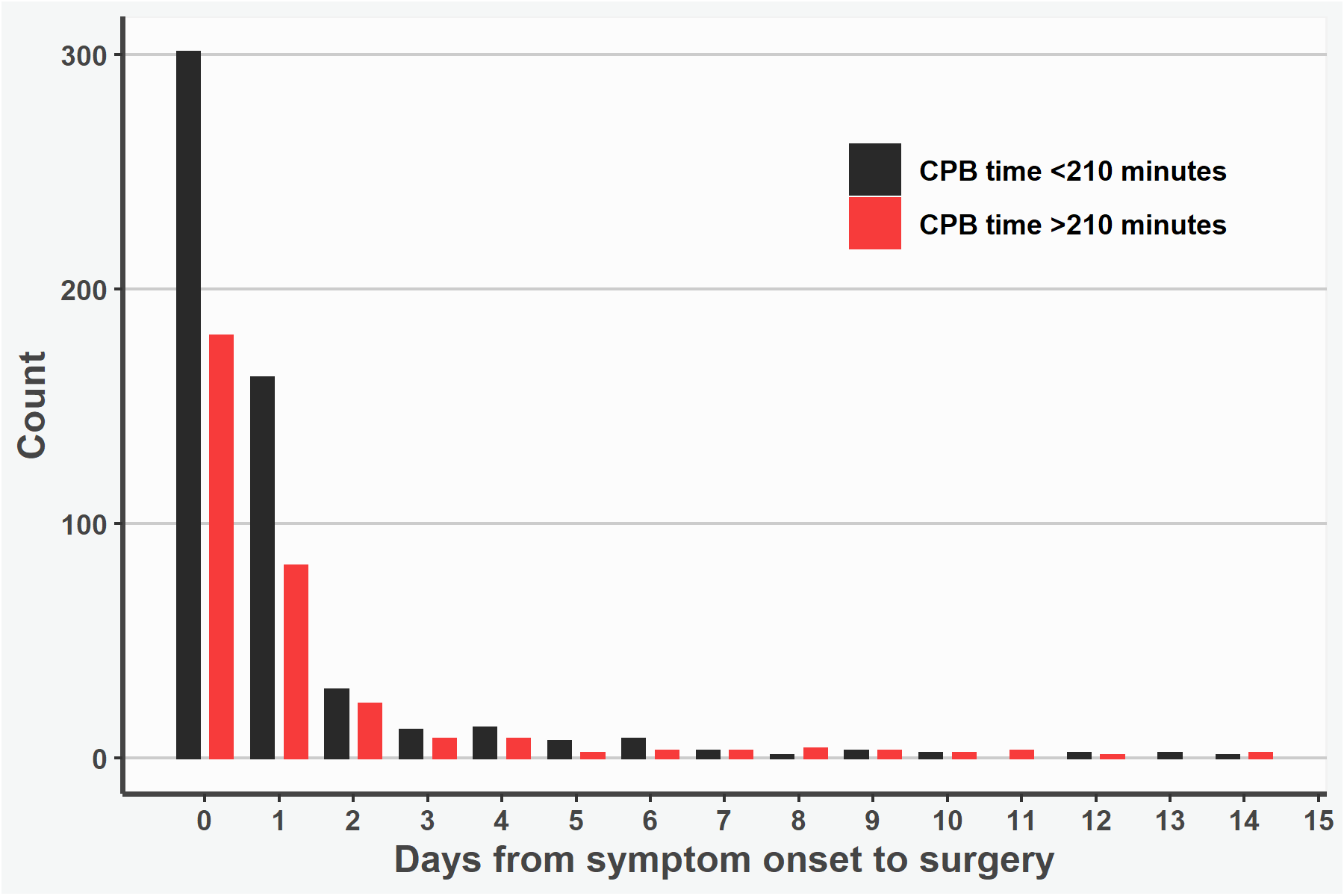

Supplement: Supplementary file 1 [file jcdd-12-00139-s001.zip › Supplementary_fig1.tiff]

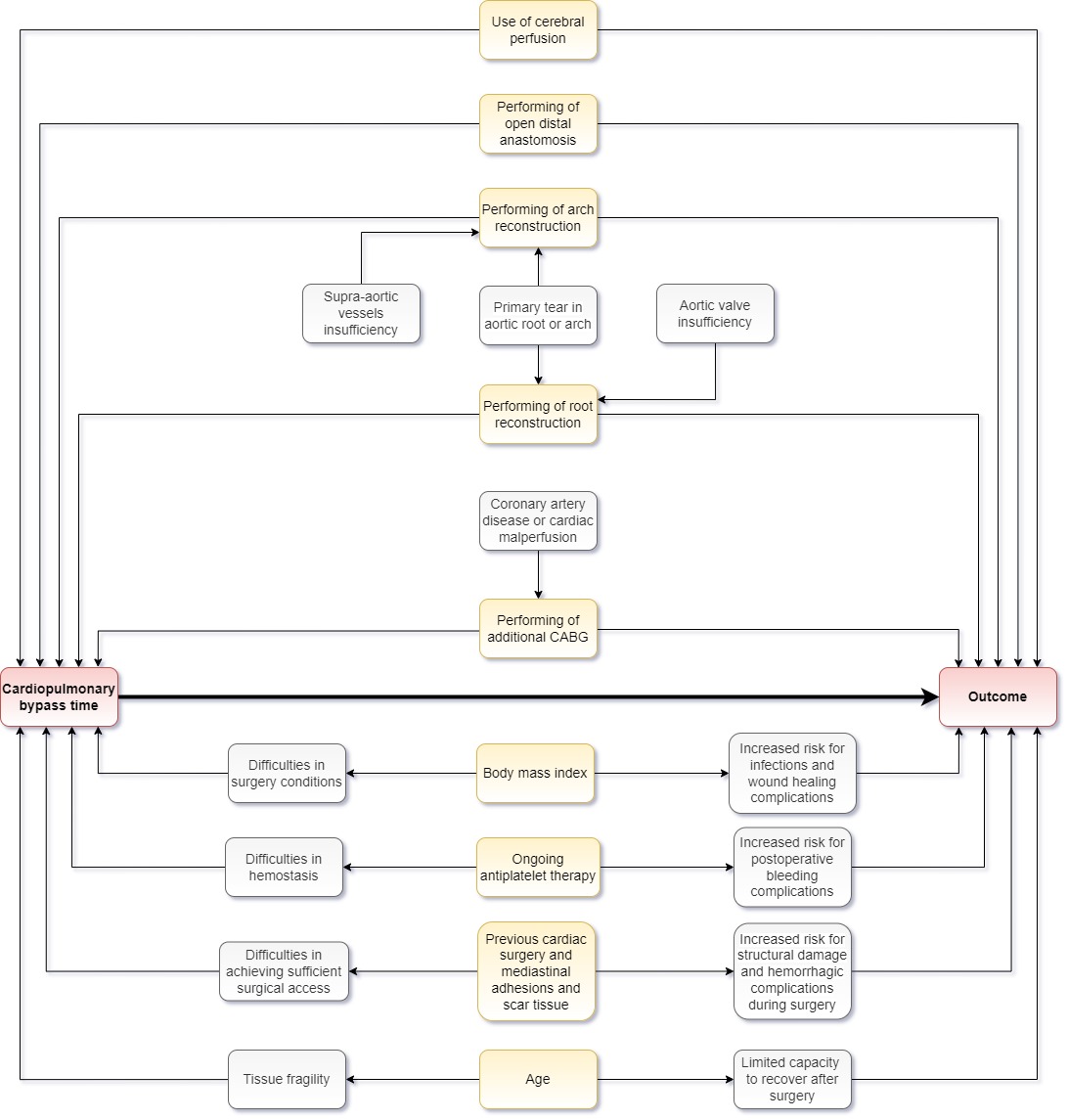

Supplement: Supplementary file 1 [file jcdd-12-00139-s001.zip › Supplementary_fig2.jpg]

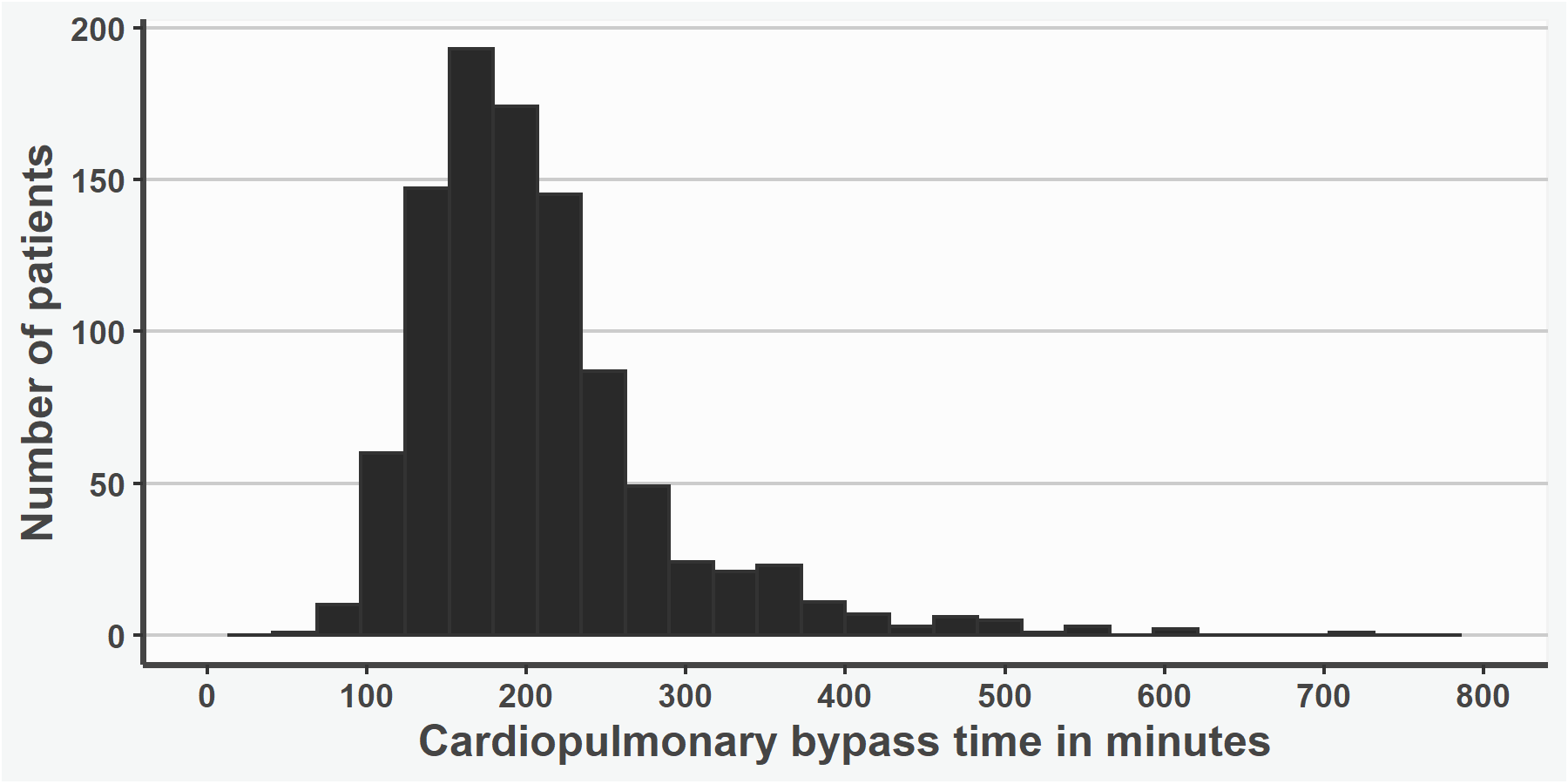

Supplement: Supplementary file 1 [file jcdd-12-00139-s001.zip › Supplementary_fig3.tiff]

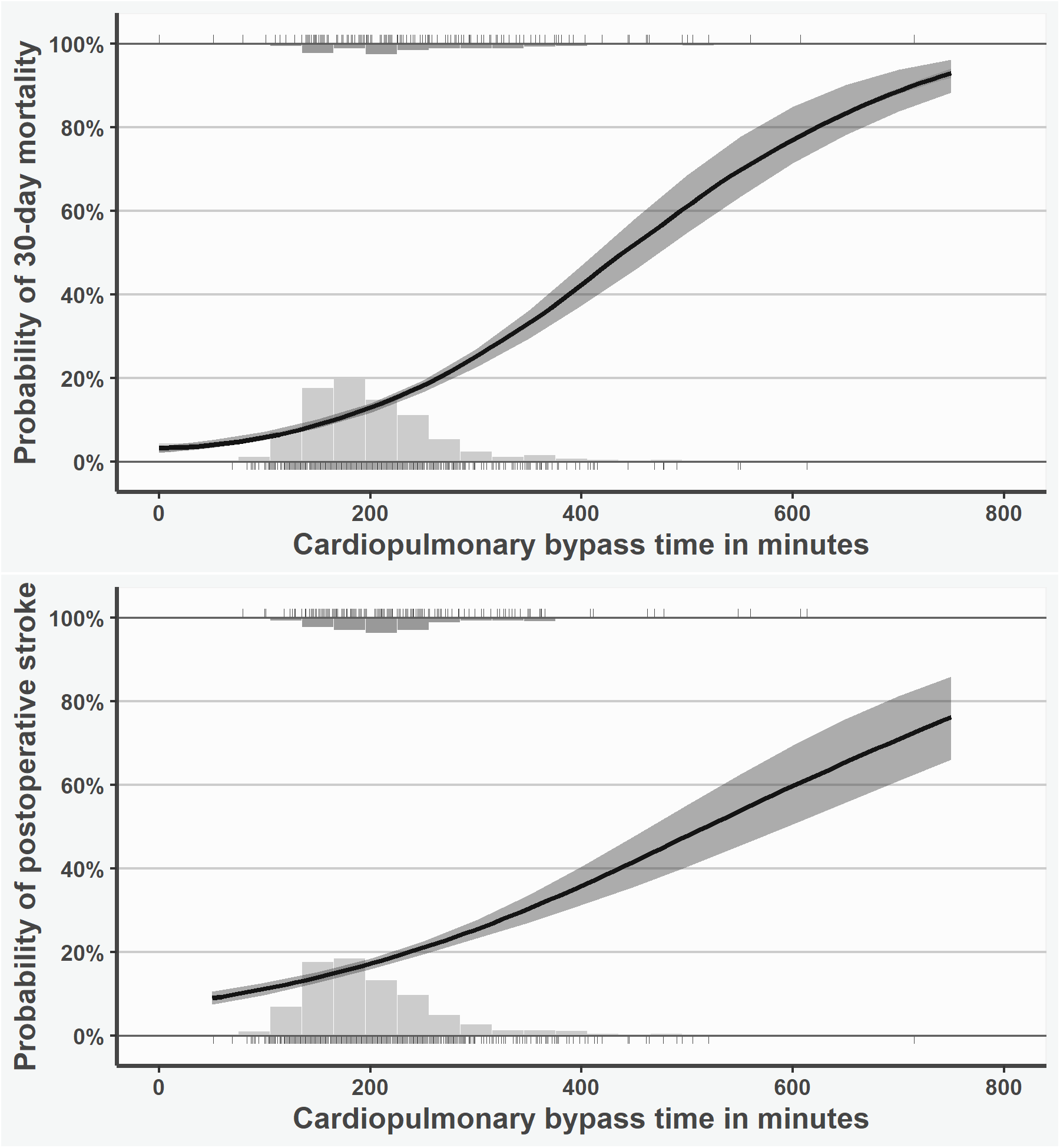

Supplement: Supplementary file 1 [file jcdd-12-00139-s001.zip › Supplementary_fig4.tiff]

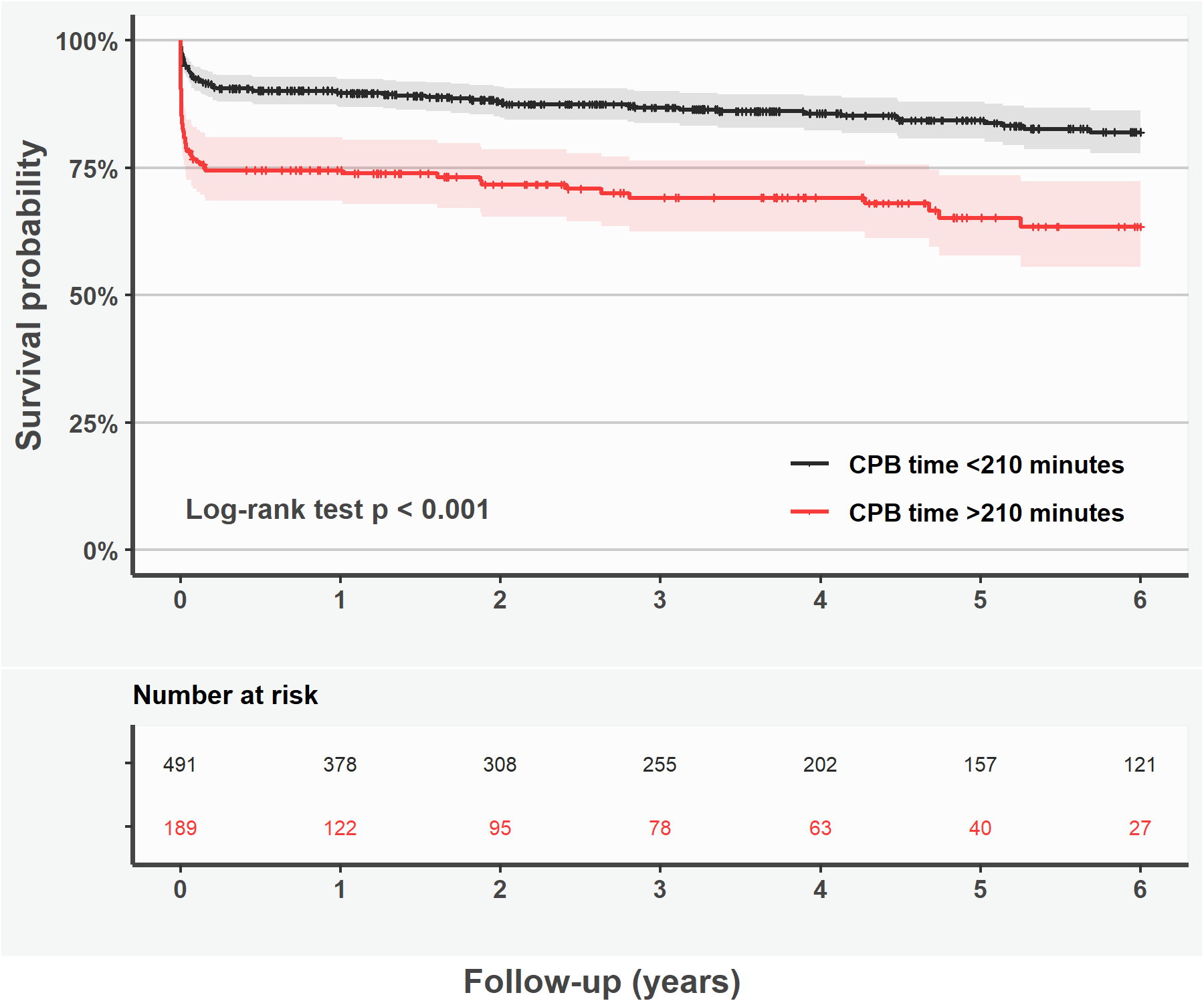

Supplement: Supplementary file 1 [file jcdd-12-00139-s001.zip › Supplementary_fig5.tiff]

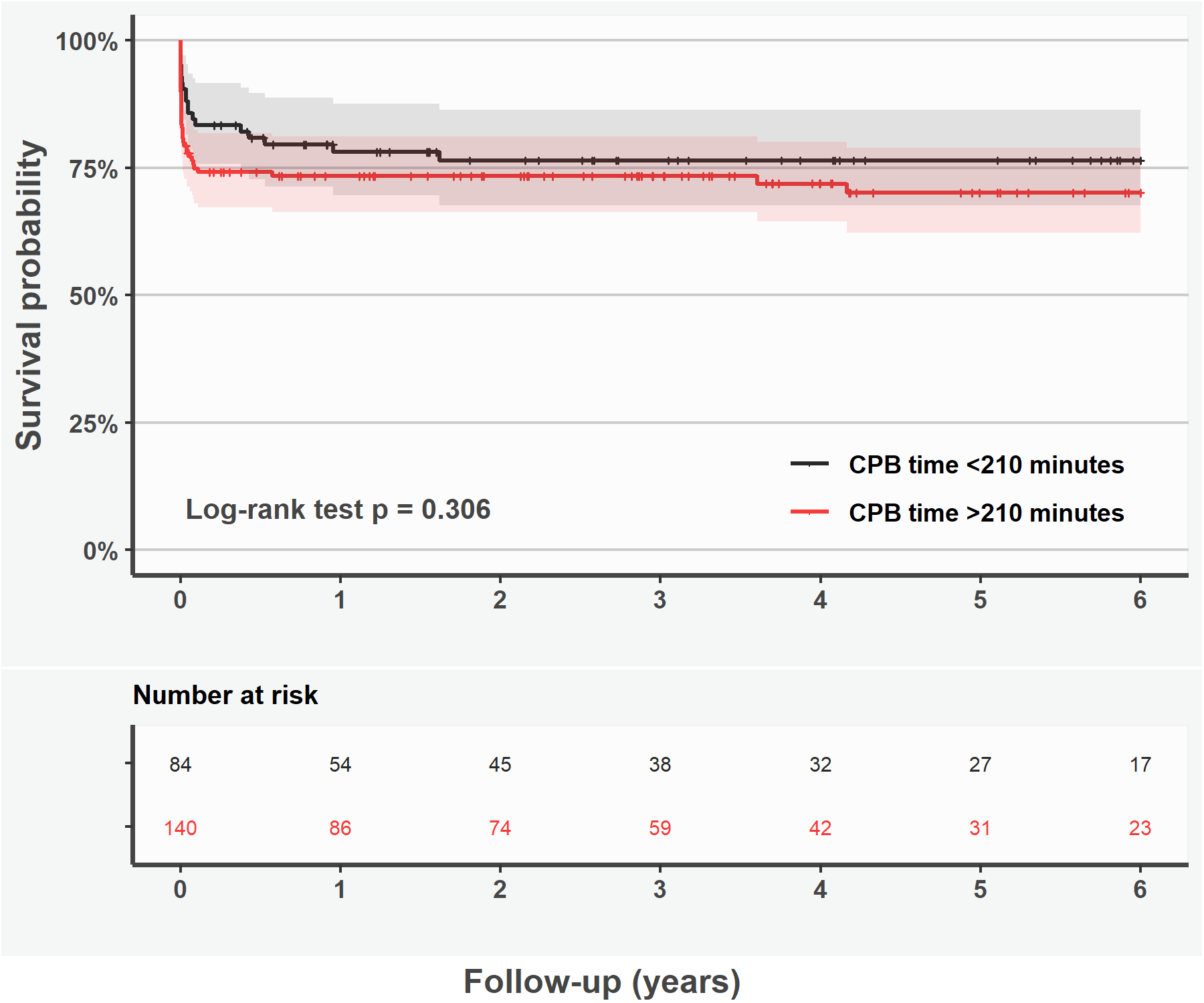

Supplement: Supplementary file 1 [file jcdd-12-00139-s001.zip › Supplementary_fig6.tiff]
